# A prospective study to evaluate the gender prediction of blastocysts by using cell-free DNA within a culture medium

**DOI:** 10.18502/ijrm.v20i7.11558

**Published:** 2022-08-08

**Authors:** Somayeh Barzanouni, Farideh Moramezi, Mahvash Zargar, Hamid Galehdari, Masoud Hemadi

**Affiliations:** ^1^Department of Obstetrics and Gynecology, Ahvaz Jundishapur University of Medical Sciences, Ahvaz, Iran.; ^2^Fertility, Infertility and Perinatology Research Center, Ahvaz Jundishapur University of Medical Sciences, Ahvaz, Iran.; ^3^Department of Genetics, Faculty of Sciences, Shahid Chamran University of Ahvaz, Ahvaz, Iran.

**Keywords:** Preimplantation diagnosis, Embryo implantation, Culture media, Blastocyst, Polymerase chain reaction.

## Abstract

**Background:**

Preimplantation genetic diagnosis (PGD) has been used as an option for couples with the possibility of having a baby with a genetic disorder. The common method for performing this test involves isolating 1 cell from day 3 or a few cells from day 5 embryos and performing genetic studies on the cell-extracted DNA. This method is invasive and can cause abortion after implantation in the uterus. Because of this, 2 noninvasive methods for performing a PGD have been studied: PGD using blastocyst fluid and PGD using embryo culture medium.

**Objective:**

The aim of this study is to determine the sensitivity of the polymerase chain reaction (PCR) technique to detect the Y chromosome using cell-free DNA within a culture medium for gender prediction of blastocysts.

**Materials and Methods:**

In this study, the gender of 30 embryos on day 5 was determined using embryonic DNA extraction from the culture medium and the PCR technique to evaluate the sex-determining region Y and fragile X mental retardation genes. Then, the accuracy was assessed using ultrasound.

**Results:**

The results of the PCR technique showed that 7 embryos were male, but an ultrasound revealed that 13 were male.

**Conclusion:**

The given results indicated that, because of the low amount of DNA extracted from the culture medium, the diagnosis of the existence of the Y chromosome by this method is still not accurate enough for detecting the gender of the embryo.

## 1. Introduction

One of the essential functions of using in vitro fertilization (IVF) techniques is to raise the possibility of having a healthy child in couples who are in danger of having a child with a genetic disease (1, 2). To accomplish this, before embryo implantation in the uterus, the genetic health of the embryo produced in laboratory conditions is examined using preimplantation genetic diagnosis (PGD). The embryo is then transferred to the uterus after assuring genetic health (1-4).

The common method of PGD implementation includes controlled ovarian stimulation, egg acquisition, intracytoplasmic sperm injection in the egg, keeping the embryos in a culture medium, detachment of 1 cell from an embryo on day 3 or a few cells on day 5 for genetic diagnosis, performing the necessary tests to diagnose, and finally selecting an embryo without disease and transferring it to the uterus (4-6). This method, unlike other diagnostic methods, does not cause complications that may lead to termination of pregnancy (3). However, this method of PGD, due to cell separation from the embryo, is a kind of invasive method so it can cause abortion after implantation (7, 8). Recently, 2 noninvasive methods for implementing PGD for obtaining embryo genetic material have been introduced, including PGD using blastocyst fluid and PGD using embryo culture medium (7-11). In both approaches, the required embryonic DNA is obtained from matters discarded after implantation in the uterus. The main drawback of both approaches is access to a small amount of genetic material in the embryo, making the performance of genetic studies difficult (7, 9). According to a study, it was estimated that 80 ± 70 and 99 ± 113 pg of DNA were present in the medium on day 3 and day 5/6, respectively. The study showed that with this amount of DNA, the TBC1 domain family member 3 gene, which is located on chromosome 17, could be identified, but it was not enough to accurately identifythe testis-specific protein Y-linked 1 gene, which is located on the Y chromosome (12). It was reported that combining the amount of DNA obtained from both methods is sufficient for whole genome amplification and accurate aneuploidy screening (9).

Due to medical reasons, gender selection is defined as preventing childbirth with a severe genetic disorder (13, 14). PGD was initially introduced for selecting female embryos concerning chromosome X-related disorders (5, 14, 15). Although, currently, some families, due to several issues such as having multiple children of the same sex and wanting to have a child of different gender, request IVF implementation and pre-implantation genetic diagnosis (16).

This prospective clinical trial study attempted to determine the gender of embryos formed in the laboratory on day 5, using embryonic DNA extraction from the culture medium, and evaluation of the sex-determining region Y (*SRY)* and fragile X mental retardation (*FMR1)* genes for diagnosis of the Y and X chromosomes, respectively, using the polymerase chain reaction (PCR) technique (17-19). Then, after the diagnosis of the gender of implanted embryos, in the 4 ${}^{\text{th}}$ month of pregnancy the accuracy of this method was evaluated by ultrasound.

## 2. Material and Methods

### Sample collection and preparation

This study was performed between December 2018 and March 2020 in Imam Khomeyni hospital, Ahvaz Jundishapur University of Medical Sciences, Ahvaz, Iran.

According to the Cochran test, the minimum number of embryos required was 23. Due to the possibility of losing samples during the study, 30 samples were examined in the study.


$$n=\frac{{Nz}^{2}pq}{{Nd}^{2}+z^{2}pq}=\frac{30\times 1.96^{2}\times 0.5\times 0.5}{30\times 0.1^{2}+1.96^{2}\times 0.5\times 0.5}=23$$

The inclusion criteria were couples referred to the fertility clinic of Imam Khomeyni hospital for infertility and seeking assisted reproductive technology and IVF assistance using PGD for determining the gender of embryos, with female partners < 35 yr old.

The exclusion criteria were couples with cancelled cooperation, unsuccessful IVF, and no pregnancy or abortion before the 4 ${}^{\text{th}}$ month of pregnancy. If the IVF was unsuccessful or the pregnancy was terminated before birth, a new couple was substituted.

Couples underwent transvaginal oocyte retrieval and fertilization using intracytoplasmic sperm injection. Following the intracytoplasmic sperm injection, fertilized oocytes were cultured in G-TL ${}^{\text{TM}}$ media (Vitrolife, Sweden) at 37ºC with 6% CO ${}_{2}$ and 5% O ${}_{2}$ . A total of 30 preimplantation embryos (calculated based on Cochran test) at day 5 were examined. After the embryo was removed, 20 μl of media was recovered from each day 5 embryo culture and transferred to DNA-free/DNase-free tubes under sterile conditions. DNA extraction and amplification were performed using the REPLI-g Single Cell Kit (QIAGEN, Netherlands) following the manufacturer's protocol. The amplification product was quantified with nanodrop. Finally, the samples were stocked at 4ºC until PCR.

### PCR assay

The primers of the *SRY* and *FMR1* genes were designed using NCBI Primer-Blast (Table I). PCR was performed based on the table II steps (according to the manufacturer's protocol) in a thermal cycler (Perkin Elmer 480). Electrophoresis of the PCR products was performed on 1.5% agarose gels for 60 min. The gel was then stained with ethidium bromide and observed under a UV transilluminator. Then, according to the following 4 states, the blastocysts' gender was predicted.

*SRY*+ / *FMR1*+, blastocyst has both X and Y chromosomes, blastocyst gender should be male.

*SRY*- / *FMR1*+, blastocyst has only an X chromosome, blastocyst gender should be female.

*SRY*+ / *FMR1*-, blastocyst has only a Y chromosome, maybe blastocyst has aneuploidy, the test should be repeated.

*SRY*- / *FMR1*-, blastocyst has neither X nor Y chromosomes, the test should be repeated.

**Table 1:** Designed primers for *SRY* and *FMRP* genes


**Gene name **	**Product length**	**Primer type**	**Sequence (5' → 3')**	**Length**	**Tm**	**GC%**	**Self-complementarity**
	Forward	CGCATTCATCGTGTGGTCTC	20	59.35	55.00	2
***SRY***	230	Reverse	AATTCTTCGGCAGCATCTTCG	21	59.33	47.62	5
			Forward	GAGATGACCAGTGTGTTCCAGT	22	59.96	50.00	3
***FMR1***	302	Reverse	CGCAGCCGACTACCTTCAC	19	60.81	63.16	4
		*SRY: *Sex-determining region Y,* FMR1:* Fragile X mental retardation, Tm: Primer melting temperature, GC%: Guanin cytosine nucleotides percentage in primer							

**Table 2:** PCR program


**Stage**	**Temperature (ºC)**	**Time (sec)**
**Polymerase activation/denaturation**	94	120
	94	15
	58	20
**Amplification (35 cycles)**	72	20
	**Final extension**	72	600
	PCR: Polymerase chain reaction		

### Ethical considerations

In this prospective study, all the clinical information was obtained from the Fertility, Infertility Private Center. All participating couples completed the informed consent form. The clinical analysis was approved by the Ethics Committee of Ahvaz Jundishapur University of Medical Sciences, Ahvaz, Iran (Code: IR.AJUMS.REC.1398.990).

## 3. Results

In this study, 117 couples participated, of which 54 became pregnant. Finally, in 30 pregnancies, the sex of the fetus was determined by ultrasound, and all pregnancies were successful, and the baby was born alive.

**Figure 1:**
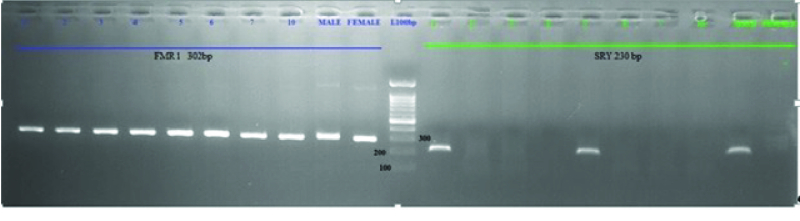
Amplification of *FMR1* (302 bp) and *SRY* (230 bp) genes from the blastocyst culture medium. The culture medium was collected from day 5, healthy male and female served as control.

The *SRY* and *FMR1* genes were successfully amplified in a day 5 culture medium and detected with bands at 230 bp and 301 bp, respectively. Positive controls (male and female DNA) were included in the PCR reactions. If PCR products were observed at both 230 bp and 301 bp, this result indicated that the DNA originated from a male sample. However, if samples originated from a female, a single PCR product at 301 bp was observed (Figure 1).

Analysis of the PCR products showed that out of the 30 embryos studied, 7 were male, and 23 were female. However, an ultrasound revealed that 13 fetuses were male and 17 were female. Thus, in 6 male embryos, the Y chromosome was not detected by the amplified *SRY* gene, and sex determination was incorrect. The calculation of the sensitivity, specificity, and diagnostic accuracy of this method was done based on the `2 by 2 table' and the results were as follows (Table III):


$$\mathrm{𝑆𝑒𝑛𝑠𝑖𝑡𝑖𝑣𝑖𝑡𝑦}\left(\%\right)=\frac{\mathrm{𝑁𝑢𝑚𝑏𝑒𝑟}\mathrm{𝑜𝑓}\mathrm{𝑡𝑟𝑢𝑒}\mathrm{𝑝𝑜𝑠𝑖𝑡𝑖𝑣𝑒𝑠}}{\mathrm{𝑁𝑢𝑚𝑏𝑒𝑟}\mathrm{𝑜𝑓}\mathrm{𝑡𝑟𝑢𝑒}\mathrm{𝑝𝑜𝑠𝑖𝑡𝑖𝑣𝑒𝑠}+\mathrm{𝑁𝑢𝑚𝑏𝑒𝑟}\mathrm{𝑜𝑓}\mathrm{𝑓𝑎𝑙𝑠𝑒}\mathrm{𝑛𝑒𝑔𝑎𝑡𝑖𝑣𝑒𝑠}}\times 100=\frac{7}{13}\times 100=53.84$$

$$\mathrm{𝑆𝑝𝑒𝑐𝑖𝑓𝑖𝑐𝑖𝑡𝑦}\left(\%\right)=\frac{\mathrm{𝑁𝑢𝑚𝑏𝑒𝑟}\mathrm{𝑜𝑓}\mathrm{𝑡𝑟𝑢𝑒}\mathrm{𝑛𝑒𝑔𝑎𝑡𝑖𝑣𝑒𝑠}}{\mathrm{𝑁𝑢𝑚𝑏𝑒𝑟}\mathrm{𝑜𝑓}\mathrm{𝑡𝑟𝑢𝑒}\mathrm{𝑛𝑒𝑔𝑎𝑡𝑖𝑣𝑒𝑠}+\mathrm{𝑁𝑢𝑚𝑏𝑒𝑟}\mathrm{𝑜𝑓}\mathrm{𝑓𝑎𝑙𝑠𝑒}\mathrm{𝑝𝑜𝑠𝑖𝑡𝑖𝑣𝑒𝑠}}\times 100=\frac{17}{17}\times 100=100$$

$$\mathrm{𝑃𝑜𝑠𝑖𝑡𝑖𝑣𝑒𝑝𝑟𝑒𝑑𝑖𝑐𝑡𝑖𝑣𝑒𝑣𝑎𝑙𝑢𝑒}\left(\%\right)=\frac{\mathrm{𝑁𝑢𝑚𝑏𝑒𝑟}\mathrm{𝑜𝑓}\mathrm{𝑡𝑟𝑢𝑒}\mathrm{𝑝𝑜𝑠𝑖𝑡𝑖𝑣𝑒𝑠}}{\mathrm{𝑁𝑢𝑚𝑏𝑒𝑟}\mathrm{𝑜𝑓}\mathrm{𝑡𝑟𝑢𝑒}\mathrm{𝑝𝑜𝑠𝑖𝑡𝑖𝑣𝑒𝑠}+\mathrm{𝑁𝑢𝑚𝑏𝑒𝑟}\mathrm{𝑜𝑓}\mathrm{𝑓𝑎𝑙𝑠𝑒}\mathrm{𝑝𝑜𝑠𝑖𝑡𝑖𝑣𝑒𝑠}}\times 100=\frac{7}{7}\times 100=100$$

$$\mathrm{𝑁𝑒𝑔𝑎𝑡𝑖𝑣𝑒𝑝𝑟𝑒𝑑𝑖𝑐𝑡𝑖𝑣𝑒𝑣𝑎𝑙𝑢𝑒}\left(\%\right)=\frac{\mathrm{𝑁𝑢𝑚𝑏𝑒𝑟}\mathrm{𝑜𝑓}\mathrm{𝑡𝑟𝑢𝑒}\mathrm{𝑛𝑒𝑔𝑎𝑡𝑖𝑣𝑒𝑠}}{\mathrm{𝑁𝑢𝑚𝑏𝑒𝑟}\mathrm{𝑜𝑓}\mathrm{𝑡𝑟𝑢𝑒}\mathrm{𝑛𝑒𝑔𝑎𝑡𝑖𝑣𝑒𝑠}+\mathrm{𝑁𝑢𝑚𝑏𝑒𝑟}\mathrm{𝑜𝑓}\mathrm{𝑓𝑎𝑙𝑠𝑒}\mathrm{𝑛𝑒𝑔𝑎𝑡𝑖𝑣𝑒𝑠}}\times 100=\frac{17}{23}\times 100=73.9$$

**Table 3:** The results of `2 by 2 table'


	**Ultrasound result**	
**PCR test result**	**Male gender (+)**	**Female gender (-)**
	**Male gender (+)**	7 (TP)	0 (FP)
**Female gender (-)**	6 (FN)	17 (TN)
PCR: Polymerase chain reaction, TP: True positive, FP: False positive, FN: False negative, TN: True negative		

## 4. Discussion

In this study, using embryo DNA extracted from the fetal culture medium on day 5, the authors tried to determine the presence or absence of a Y chromosome for gender determination of the studied embryo, and the sensitivity of this method was examined after the gender determination of embryos by ultrasound in the 4 ${}^{\text{th}}$ month of pregnancy. The results demonstrated that despite the easy diagnosis of the X chromosome by the *FMR1* gene, the Y chromosome diagnosis by the *SRY* gene was not as easy, so that the PCR studies identified only 7 embryos with a Y chromosome, while the ultrasound results showed the existence of 13 boys.

As a result, it can be said that although the gender of all 7 cases diagnosed by ultrasound was the same as found in the culture medium, the gender of the diagnosed girls had an accuracy of 74%. So, in case a male gender is desired, we can trust the method to determine the gender of the embryo. However, if the preferred gender is female, it should be considered that 26% of the cases diagnosed as females using this method are probably males.

One of the factors that can explain this result is the differences in the size and dimensions of the 2 chromosomes X and Y. The Y chromosome is one of the smallest human chromosomes with approximately 57.23 nucleotides, and the *SRY* gene, located at the Yp11.2 locus, comprises only 828 nucleotides. In contrast, the X chromosome is about 156.05 Mbp long (2.72 times the Y chromosome), and the *FMR1* gene, located at locus Xq27.3, has 46,135 nucleotides (about 55.71 times larger than the *SRY* gene).

A previous study used multiplex PCR for the detection of *SRY* (as a Y chromosome gene) and *ALT1* genes (as a control). This study was performed on DNA extracted from blood cells. Its purpose was to evaluate the efficiency of the multiplex PCR technique and the sensitivity of the *SRY* gene to detect the male gender (18).

Another study reported that the use of cell-free DNA in the culture medium may be a viable alternative to the use of embryonic cell DNA for the diagnosis of a-thalassemia-SEA (20). The study evaluated the amount of cell-free DNA in the culture medium from the culture on day 3 and day 5, and reported that it was better to use the day 5 medium due to the higher amount of cell-free DNA.

Furthermore, Li et al. showed that cell-free DNA in a culture medium or fetal blastocyst fluid could be used to assess genetic and chromosomal defects. However, the amount of DNA obtained from these methods is small, and it may be necessary to use both ways together (7).

According to Yang et al., the amount of cell-free DNA in the day 3 embryonic culture medium was insufficient to detect the Y chromosome by the *SRY* gene, and it was recommended to use the day 5 embryonic culture medium (8).

Given the huge difference in the dimensions of these 2 chromosomes, it is clear that the extraction of the Y chromosome and the study of its genes is more difficult than the extraction and study of the genes of the X chromosome, particularly in the extraction of an embryo's genetic material from the culture medium where the amount of DNA obtained for examination is small.

Our findings are consistent with a study that showed that the amount of DNA obtained from culture media was not sufficient for accurately detecting the testis-specific protein Y-linked 1 gene, which is located on the Y chromosome, but was enough for detecting the TBC1 domain family member 3 gene, which is located on chromosome 17 (12).

## 5. Conclusion

The findings of this study suggest that simultaneous extraction of fetal DNA from the blastocyst fluid and from the embryo culture medium enhances the amount of fetal DNA obtained for examinations. Also, for increasing the accuracy of Y chromosome detection and detecting the *SRY* gene, some of the unique sequences of this chromosome should be examined simultaneously using the multiplex PCR technique.

##  Conflict of Interest

The authors declare that there is no conflict of interest.
